# Ni–Cr Powders Modified with Rhenium as a Novel Coating Material—Physical Properties, Microstructure, and Behavior in Plasma Plume

**DOI:** 10.3390/ma15113844

**Published:** 2022-05-27

**Authors:** Adriana Wrona, Marcin Lis, Krzysztof Pęcak, Izabela Kalemba-Rec, Stanisław Dymek, Mirosław Wróbel, Katarzyna Bilewska, Katarzyna Kustra, Marek Stanisław Węglowski, Piotr Śliwiński

**Affiliations:** 1 Łukasiewicz Research Network—Institute of Non-Ferrous Metals, Sowińskiego Str. 5, 44-100 Gliwice, Poland; adriana.wrona@imn.lukasiewicz.gov.pl (A.W.); marcin.lis@imn.lukasiewicz.gov.pl (M.L.); katarzyna.bilewska@imn.lukasiewicz.gov.pl (K.B.); katarzyna.rebisz7@gmail.com (K.K.); 2 Faculty of Metals Engineering and Industrial Computer Science, AGH University of Science and Technology, Czarnowiejska Str. 66, 30-054 Kraków, Poland; kalemba@agh.edu.pl (I.K.-R.); gmdymek@cyfronet.pl (S.D.); mwrobel@agh.edu.pl (M.W.); 3 Łukasiewicz Research Network—Institute of Welding, Bł. Czesława Str. 16-18, 44-100 Gliwice, Poland; marek.weglowski@is.lukasiewicz.gov.pl (M.S.W.); piotr.sliwinski@is.lukasiewicz.gov.pl (P.Ś.)

**Keywords:** nickel alloys, rhenium, phase analysis, microstructure, plasma spraying, refractories, nonferrous metals, coatings, thermal spraying, powder metallurgy

## Abstract

The aim of this work was to develop a new coating material based on Ni20Cr alloy modified with up to 50%wt. rhenium. The modification was carried out by the mechanical mixing of the base powder and ammonium perrhenate with the subsequent thermoreduction in an H_2_ atmosphere. The obtained powder consists of a nickel–chromium core surrounded by a rhenium shell. The characterization of the powders—including their microstructure, phase and chemical composition, density, flowability, particle size distribution, and specific surface area—was performed. The influence of plasma current intensity and hydrogen gas flow on in-flight particle temperature and velocity were investigated. The results indicate that there is interdiffusion between the base Ni20Cr and the rhenium shell, resulting in intermediary solid solution(s). The modified powders have a higher specific surface area and a lower flowability, but this does not prevent them from being used as feedstock in plasma spraying. In-flight measurements reveal that increasing the content of rhenium allows for the higher temperature of particles, though it also reduces their speed.

## 1. Introduction

Corrosion and erosion are major problems in a variety of industrial applications. Their direct result is degradation and deterioration of industrial equipment, affecting not only production costs, but also workplace safety.

One of the ways to prevent and limit the effects of corrosion and erosion is the use of protective coatings. Nickel-based coatings containing chromium are often utilized for this purpose due to their high-temperature oxidation resistance [1,2,3,4,5]. Ni–20Cr coatings are commonly applied to improve the degradation resistance of boilers used in energy sectors [3,6]. There are many grades of Ni–Cr alloys containing other elements such as Mo, Nb, Fe, and Ti that improve the physicochemical properties of these alloys. The most widely used alloys are commercial Inconel 625 and Inconel 718.

Rhenium is a precious and rare metal. Its melting point is 3186 °C, which is the third highest melting point of all the elements. This, together with its high boiling point (5596 °C), high density (21.03 g/cm^3^), and high hardness and resistance to corrosion and deformation, makes rhenium unique. Furthermore, rhenium exhibits the third highest Young’s modulus among all elements at 461–471 GPa. In addition, it has very good abrasion properties, a high tensile strength, and a creep resistance up to 2000 °C. It also exhibits good corrosion resistance in seawater, as well as hydrochloric and sulfuric acids [7].

Rhenium is mostly used as an alloying element in nickel-based superalloys typically utilized in the aerospace industry for turbine production. Rhenium addition to nickel superalloys improves their high-temperature strength and creep resistance [8,9]. Rhenium reinforces the γ phase of the superalloy and has a beneficial effect on its microstructure, resulting in a good creep resistance at high temperatures [10,11,12]. Rhenium’s beneficial effect on the microstructure of Co–Ni alloys was also reported [13]. Products made of nickel-based superalloys are usually manufactured by forging and casting. Most often, conventional casting (CC), directional solidification (DS), and single crystal casting (SC) methods are used. Modern superalloys containing rhenium are manufactured with the single crystal casting method.

Rhenium is also noted for improving the ductility of other refractory metals, such as tungsten and molybdenum. This effect was described as early as 1955, when it was reported that a 35% addition of rhenium to tungsten allows for rolling at room temperature [14,15]. Alloying tungsten with rhenium significantly reduces its ductile-to-brittle transformation temperature (DBTT) [16]. Rhenium also improves the creep resistance of tungsten alloys by promoting grain growth [17]. The beneficial influence of rhenium on tungsten and molybdenum alloys, comprising the simultaneous enhancement of strength, ductility, and weldability, as well as the lowering of ductile-to-brittle transition temperature, is often referred to in the literature as a “rhenium effect”. Tungsten–rhenium alloys are often used in the nuclear and aerospace industries [16].

The concept of modifying nickel-based alloys dedicated for coating processes with Re is a novel approach not previously explored. Recently, there have been some reports that suggest the feasibility of modifying coating materials with rhenium and the subsequent need to investigate such materials. For example, Cr-Al_2_O_3_ alloyed with a 5 vol.% Re addition was investigated [18]. The authors reported an increase in hardness compared to the non-modified material. Composite coatings (Mo,Re)-(Mo,Re)Si2 prepared on a ceramic substrate were reported as suitable for use in the glass production industry [19,20]. Research was also performed on the modification of AlSi9Mg alloy with rhenium subjected to friction stir processing (FSP). An increase in microstructural hardness was reported for areas enriched with rhenium [21]. Rhenium was also investigated in relation to laser remelting processes [22].

The aim of this research was to develop a new coating material based on the Ni20Cr powder whose particle surface is modified with rhenium. The authors believe that this modification could result in an improvement in the physicochemical properties and microstructure of protective layers produced from such a manufactured powder. The authors expect similar results as for Ni20Cr powders modified with 20 vol.% Mo [23]. In the referenced work, molybdenum was used to create a layer on Ni20Cr powders by mechanical milling. This was done to achieve higher temperatures of in-flight particles during the plasma spraying process. The authors assume that the higher in-flight particle temperature will result in a superior metallurgical bond between the coating and base material, as well as between the particular coating interlayers. The influence of molybdenum on the in-flight temperature of particles was indirectly determined by the microstructural and property evaluation of the prepared layers, as well as by numerical modeling performed in accordance with the method described in Ref. [24]. The calculations showed that powders coated with molybdenum could be heated in a plasma plume to a temperature approximately 600 °C higher than for unmodified powders. In this case, it also means that the powders could achieve sufficient temperatures to melt molybdenum and thus facilitate the alloying of Ni20Cr and Mo. Microstructural analysis of the produced coatings show that particles achieved high enough temperatures to induce metallurgical bonding between the base material and the first layer of the coating as well as between particular interlayer lamellae. The coatings made from the Mo-modified powder were less porous, had a better mechanical strength, and a higher oxidation resistance than those made from conventional, unmodified Ni20Cr alloy.

The influence of in-flight particle temperatures on coating properties was most extensively described in reference to ceramic materials [25,26,27,28]. The change in torch current from 350 to 650 A was reported to have increased the temperature of LaMgAl_11_O_19_/YSZ particles by approximately 300 °C, resulting in a higher hardness (an increase from ~500 to 880 HV) and a higher elastic modulus (an increase from ~65 to 125 GPa) of the coatings [25]. Other authors reported that for such materials as Al_2_O_3_-CNT and Ti_3_SiC_2_, an increase in torch power results in the increase in in-flight temperatures and the velocity of particles [26,28]. Consequently, the produced coatings were denser and exhibited a higher hardness. Similar results were achieved for TiB_2_-Al_2_O_3_ powders [27].

In the current work, the authors analyzed the effect of rhenium modification of Ni20Cr powders on phase composition, physical properties, temperature, and velocity of in-flight particles during the atmospheric plasma spraying (APS) process.

## 2. Materials and Methods

Commercial Ni20Cr powder (AMPERIT 250, Höganäs, Sweden) with a spheroidal morphology dedicated for thermal spraying was used for preparation of the alloyed Ni20Cr + xRe powder with rhenium content (x), ranging from 10 to 50 wt.%. The basic powder properties are shown in Table 1. Ammonium perrhenate (99.99% purity, KGHM Metraco, Legnica, Poland) was used as a source of rhenium in powder modification.

To modify the base powders’ surface with metallic rhenium, thermal reduction of rhenium perrhenate in a reducing atmosphere (H_2_) was carried out.

Initially, ammonium perrhenate was milled in a ball mill to reduce the particle size to promote homogenous mixing with other components. Then the Ni20Cr powder was mixed with previously prepared ammonium perrhenate in a ball mill that resulted in Ni20Cr particles coated with ammonium perrhenate. Finally, the thermal reduction in a pure hydrogen atmosphere was carried out at 850 °C for 1 h.

Phase composition was investigated by X-Ray Diffraction (XRD), and phase identification was carried out with use of PDF4+ database by ICDD. Cell parameters of the main phases were calculated with the use of Rietveld refinement technique.

Morphology, as well as qualitative and quantitative analysis, was performed on an EPMA JXA 8230 microanalyzer (JEOL Ltd., Tokyo, Japan). Energy dispersive spectroscopy (EDS) was employed to investigate the chemical composition of powder surfaces and to create elemental composition maps (the maps were acquired at 15 kV). Wavelength dispersive spectroscopy (WDS) was used to carry out chemical quantitative analysis that was performed on powder particles cross-sections. For this purpose, a small portion of the powder was embedded in a resin and ground to reveal flat sections of powder particles.

Particle size distribution analysis (d_10_, d_50_, d_90_) was carried out by laser method, using Analysette 22 Nanotec apparatus (Fritsch, Idar-Oberstein, Germany). Specific surface area was measured with the gas adsorption method (BET) on a Gemini 2360 apparatus (Micromeritics, Norcross, GA, USA). The density of the powders was determined by a pycnometer Accupyc 1340 (Micromeritics, Norcross, GA, USA). The flowability was tested in accordance with PN EN ISO 4490, by measuring the time during which a 50 g mass of powder has flown through a standardized Hall funnel.

In-flight particle parameters were measured with an Accuraspray 4.0 (Tecnar, Saint-Bruno-de-Montarville, QC, Canada). This device allows for real-time thermal spraying monitoring. The Accuraspray 4.0 is equipped with a measuring head that allows for the measurement of particle temperature above a threshold of 1000 °C with the 3% precision range, as well as particle velocity in the range of 5 to 1200 m/s with a 2% accuracy precision range. For this purpose, a plasma torch F4 and AP-50 plasma spray system (FST, Duiven, The Netherlands) was used. These parameters were measured for every powder batch with varying torch current and plasma gas (hydrogen or argon) flowrate. The process parameters combinations are collected in Table 2.

## 3. Results

### 3.1. Phase Composition, Microstructure, and Chemical Analysis

**Figure 1:**
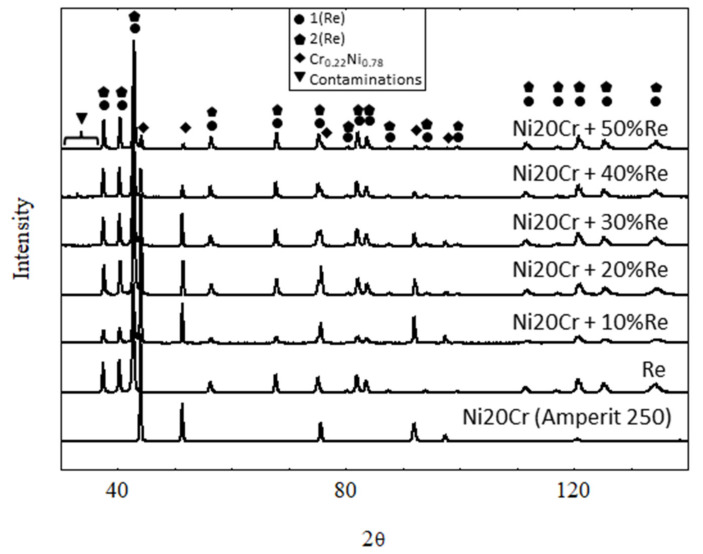
XRD diffraction pattern: NiCr (Amperit 250), rhenium and Ni20Cr + xRe powders.

**Figure 2:**
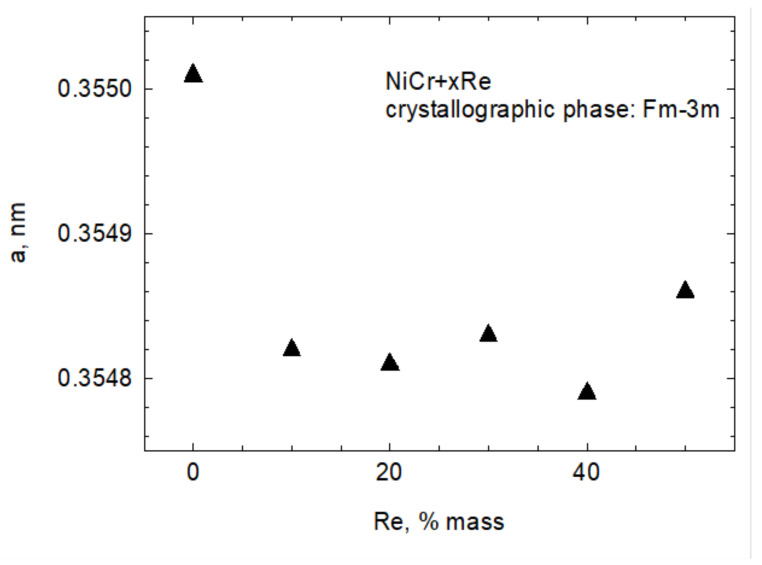
Changes in a cell parameter in relation to rhenium content in powder.

**Figure 3:**
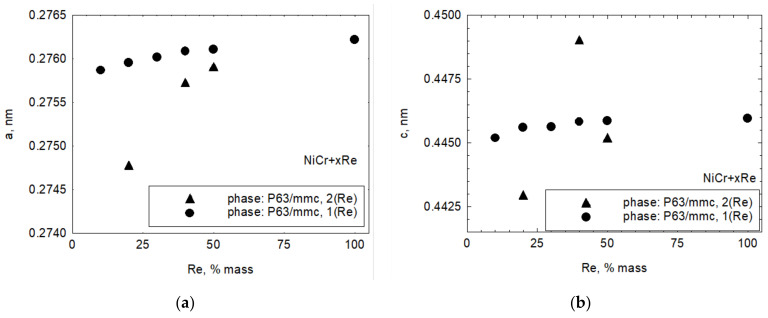
(**a**) Changes in a cell parameter in relation to rhenium content in powder, (**b**) changes in cell parameter in relation to rhenium content in powder.

Figure 1 shows the diffraction patterns of Ni20Cr + xRe (where x = 10, 20, 30, 40, 50 wt.%), Ni20Cr base alloy (AMPERIT 250), and rhenium obtained from the thermoreduction of ammonium perrhenate. The main components of the Ni20Cr + xRe powders constituted a regular Fm3m space group phase characteristic for Cr_0.22_Ni_0.78_ (card number 04-019-8411) and a hexagonal P63/mmc phase based on rhenium (card number 04-004-4396). Compared to phases identified in pure Ni20Cr and Re samples, the peaks are shifted, which may indicate a solid solution formation between the components. Apart from the two primary phases, a small but noticeable amount of other phases are present. Those phases are regarded as impurities and their XRD patterns correspond best to the Re_2_NiO_8_(H_2_O)_4_ (card number 04-010-2286) and Re_2_NiO_8_ (card number 04-009-8044) phases. The Rietveld method was used to calculate cell parameters for the main phases; however, due to the presence of other phases, the results of those calculations should be treated with care. The Bragg R factor and Rf factor for key phases did not exceed 10. For the purposes of calculations, it was assumed that Ni20Cr + xRe powders consist of two phases: a regular Fm3m (225) associated with the Ni–Cr alloy and a hexagonal P63/mmc (194) phase associated with the rhenium (labeled as 1(Re)). For Ni20Cr + xRe powders with x = 20, 40, 50, the shape of the main hexagonal phase peaks indicates they are composed of more than one constituent peak. Therefore, there are two phases of hexagonal P63/mmc structure. Due to a slight shift in the position of the second hexagonal phase it is likely that it corresponds to a solid solution of rhenium with another element, the solid solution labeled as 2(Re) (card number 01-085-7871) in this work. Figure 2 shows changes in the cell parameter a of the NiCr phase. It shows that this parameter is much smaller compared to a powder that does not contain rhenium. Figure 3 shows the parameters a and c of both hexagonal phases in reference to rhenium content. The cell parameters a and c increase slightly for phase 1(Re) with rhenium content. The parameter a of phase 2(Re) increases notably with increasing rhenium content. For this phase, the parameter c takes the highest value for x = 40%, but it should be pointed out that this sample is highly contaminated with phases Re_2_NiO_8_ and Re_2_NiO_8_(H_2_O)_4_. This could influence the results—the Bragg R factor for this sample was 14. It was necessary to include the trigonal Re_2_NiO_8_ phase in calculations for phase 2(Re).

The observed changes in cell parameters indicate that diffusion processes occur during the reduction of ammonium perrhenate between newly formed Re and the Ni20Cr base powder, resulting in a solid solution formation.

The classical chemical analysis reveals that the actual rhenium content in powders differs from the targeted one by up to 2 wt.%. Results of the analysis are shown in Table 3. A lower rhenium content can be explained by the evaporation of low melting point rhenium oxides that are likely formed during the reduction of ammonium perrhenate [29,30].

**Figure 4:**
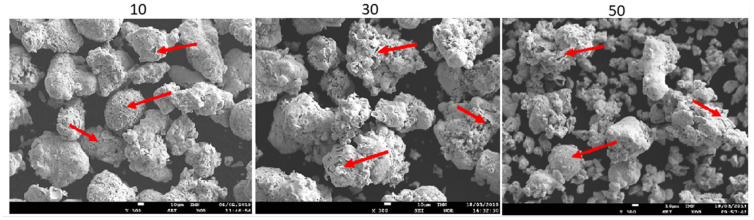
SEM pictures of powders Ni20Cr + xRe (x = 10, 30, 50 wt.%).

**Figure 5:**
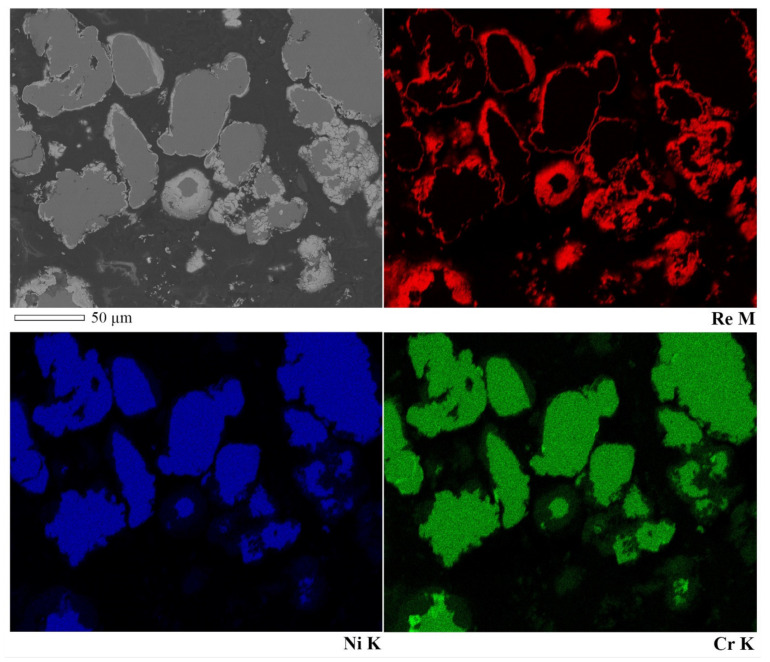
EDS map of cross-sections of NiCr + 20Re powder.

Figure 4 shows SEM images of powders modified with 10, 30, and 50 wt.% rhenium. Powders with a lower Re content consist mostly of a coarser fraction with particle sizes in the range of tens of µm. A smaller fraction with sizes of a few µm is also present but in a much smaller quantity. On the other hand, the powder containing 50 wt.% rhenium exhibits a much higher share of fine fraction. There are cracks and inhomogeneities visible on the particle surfaces. The elemental composition maps shown in Figure 5 reveal that despite those defects, the distribution of all elements is uniform. This suggests that rhenium entirely coats the particles’ surfaces, even though the thickness of rhenium layers varies from point to point, as shown in Table 4. The obtained results are similar to those in ref. [23], in which the powder also was entirely covered by the refractory shell, but the thickness of the shell varied, although the base powder used in current work was more irregular in shape.

**Figure 6:**
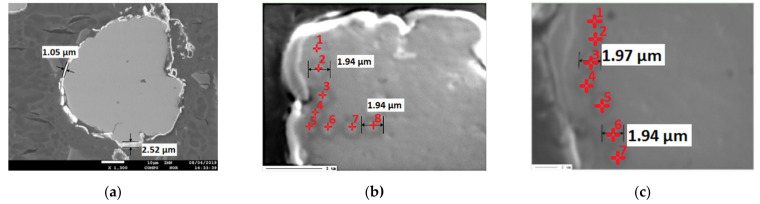
(**a**) SEM picture of NiCr + 30Re powder; (**b**) and (**c**) SEM pictures of NiCr + 30Re powder particles with marked analysis points and “burned” traces indicating excitation areas.

Elemental composition on cross-sections of the powders was investigated to verify if rhenium actually diffuses into the base material. Representative results on the example of NiCr + 30Re powder are shown in Figure 6 and Table 5. In some points near the coating, rhenium content of a few wt.% was found. Taking into account that excitation of the sample takes place in an area and not at a point, it could be possible that the signal comes from the coating and is not a result of rhenium diffusion into the NiCr alloy. To ensure the validity of the findings, marks were deliberately “burned” by extensive exposure to an electron beam at each of the testing points to show the area of excitation. Some of the analyzed points (e.g., nr 2, 4, and 5) are sufficiently distanced from the rhenium coating to rule out that the coating itself is influencing the results. This validates that Re diffused into the Ni20Cr base alloy as suggested by the XRD results. Due to the thickness of the coating in range of 1–2 µm and the size of the excitation area, it was not possible to investigate with EPMA whether or not NiCr alloy components diffused into the coating.

### 3.2. Physical Properties of the Powders

**Figure 7:**
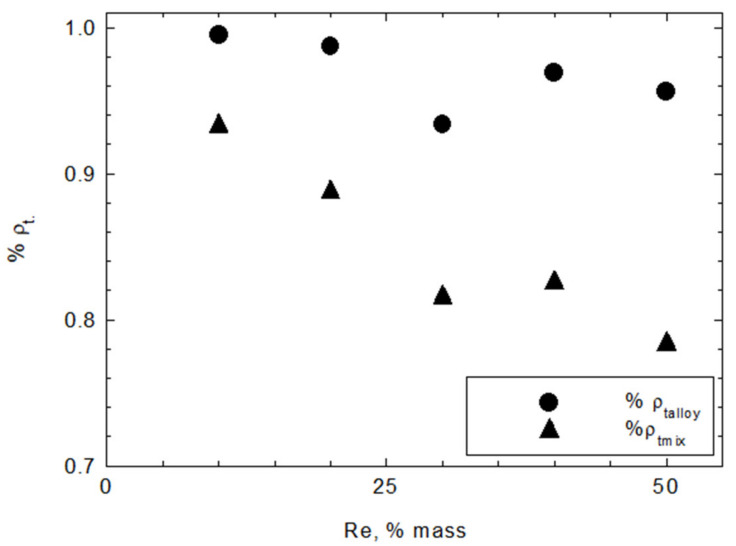
Ratio between measured density of Ni20Cr + xRe powders and values calculated based on mixture rule (mix) and additive effects of components in alloys (alloy).

Figure 7 shows the relationship between the density measured by the pycnometer method and that calculated theoretically from the mixture rule ρ_tmix_, also for alloys with the additive effect of components ρ_talloy_.

The measured density to the theoretical ρ_tmix_ density ratio decreases with increasing rhenium content in powder. The Ni20Cr + 10Re powder density was at 93% of the theoretical mixture density but falls to around 80% of this value with increased rhenium content. Using ρ_talloy_ as a reference value shows a lower discrepancy between the theory and the measurements. Powders achieved a density at a level of 94% ρ_talloy_ or higher. The results may suggest that the NiRe solutions are likely formed in the material. The lower density of produced powders may result from oxidation or closed porosity. The latter is particularly possible at the Ni20Cr and Re coating interfaces.

**Figure 8:**
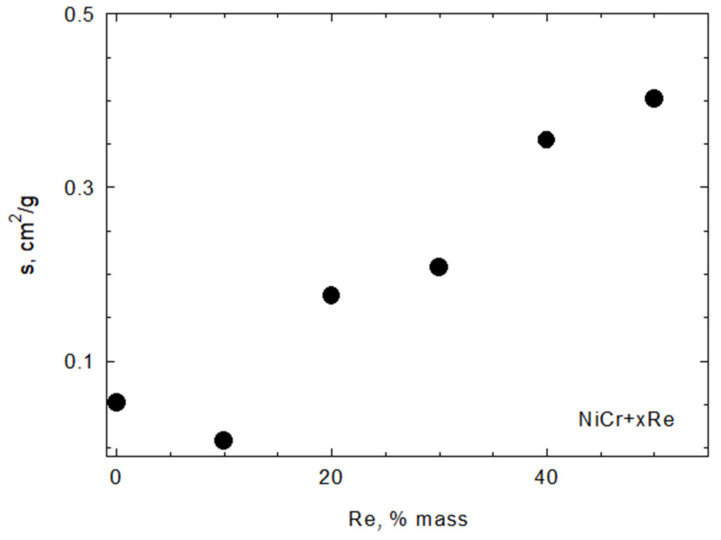
Specific surface area of NiCr + xRe powders.

**Figure 9:**
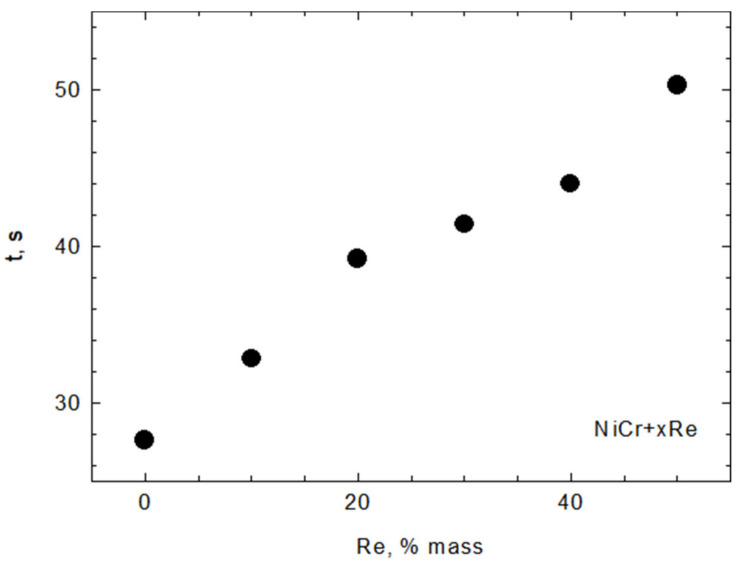
Flowability of NiCr + xRe powders.

**Figure 10:**
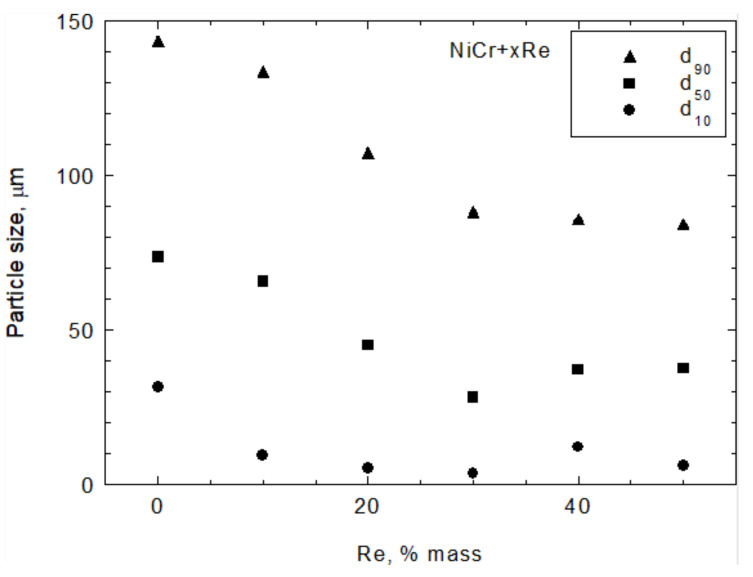
Average particle size for d_10_, d_50_, and d_90_ fractions of Ni20Cr + xRe powders.

The specific surface area of composite powders increases with the rhenium content (Figure 8) as a result of rhenium modification. However, modification also decreases the flowability of the powder, as shown in Figure 9. The particle size is also affected by modification—it decreases with increasing rhenium content of up to 30 wt.% and stabilizes with a higher Re content. The variations in particle size parameters d_10_, d_50_, and d_90_ are shown in Figure 10.

### 3.3. In-Flight Particle Properties

**Figure 11:**
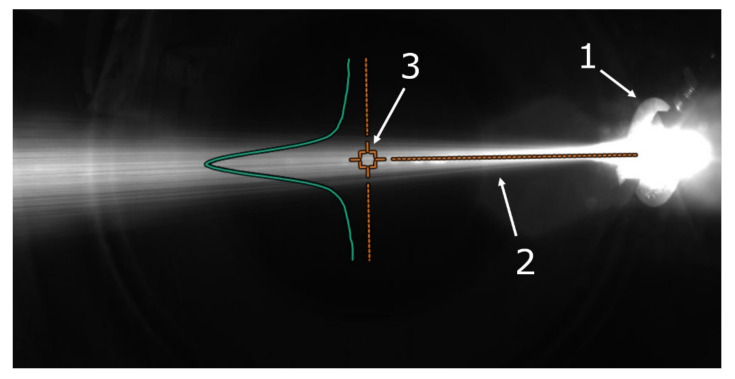
Camera view of Accuraspray 4.0 system. 1—plasma torch, 2—plasma plume, and 3—area of temperature and velocity measurement.

**Figure 12:**
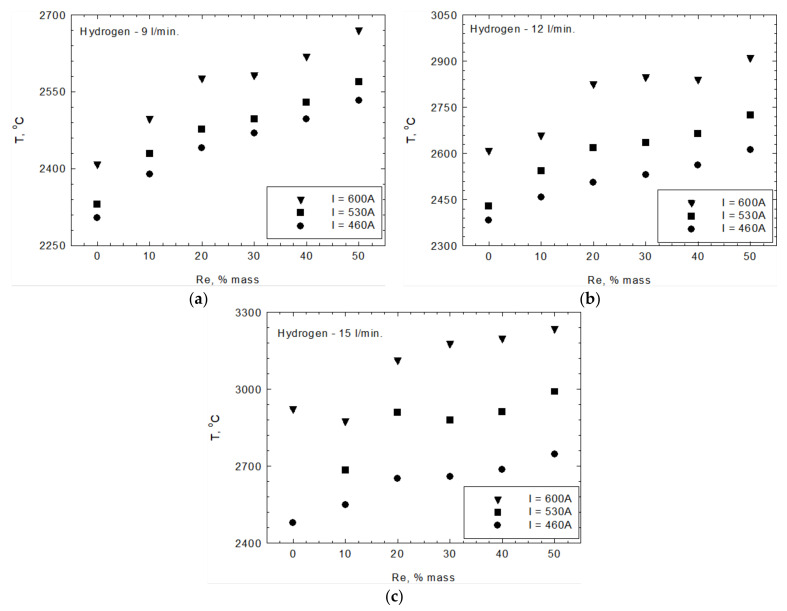
Temperature T of in-flight NiCr + xRe particles measured with current intensity (**a**) I = 460 A, (**b**) I = 530 A, and (**c**) I = 600 A.

**Figure 13:**
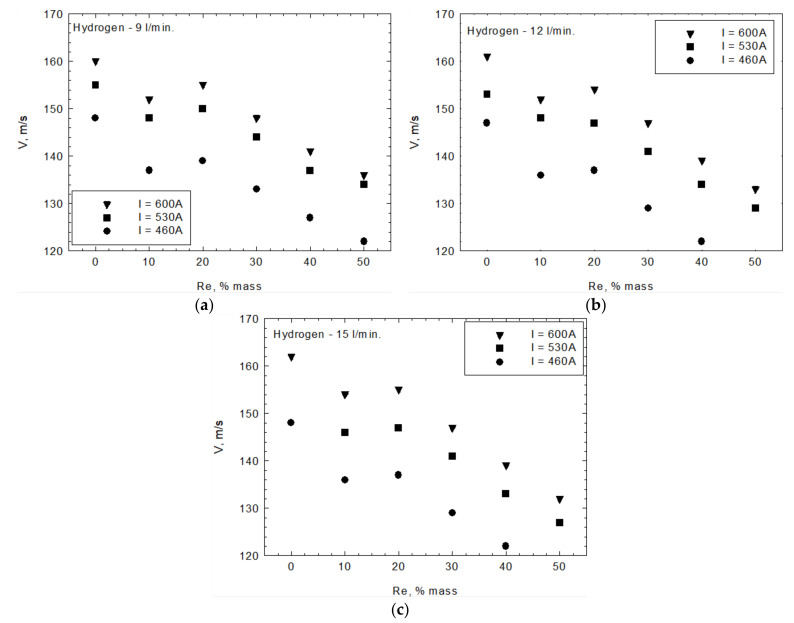
Velocity V of in-flight NiCr + xRe particles measured with current intensity (**a**) I = 460 A, (**b**) I = 530 A, and (**c**) I = 600 A.

The in-flight particle properties were analyzed by an Accuraspray 4.0 system camera. Figure 11 shows an exemplary photo of the process. The camera allows for measurements of the temperature and speed of the particles. The region where these measurements were performed is marked in Figure 11 as 3. Figure 12 and Figure 13 show the relationship between temperature, T, velocity, V, and rhenium content in the used powders. The measurements were carried out for three current intensities (I = 460, 530, 600 A) and the hydrogen flowrate (ν = 9, 12, 15 L/min). Both of those parameters influence plasma temperature. At a hydrogen flowrate of ν = 9 L/min, the temperature of the particles increases with rhenium content regardless of the current intensity used. At a hydrogen flowrate of ν = 12 L/min and a current of I = 460 A, a similar trend was observed. For higher currents of 530 and 600 A, an increase in temperature occurs up to 20 wt.% rhenium, and for even higher Re content it stabilizes. Such a behavior was also identified for ν = 15 L/min and I = 460 A parameters. With a combination of the highest hydrogen flowrate ν = 15 L/min and highest current intensity I = 600 A, there is a different relationship between the temperature of the particles and their rhenium content. In this case, for powders with 10 wt.% Re a slight decrease in temperature was observed, followed by a systematic increase for powders with a higher rhenium content.

**Figure 14:**
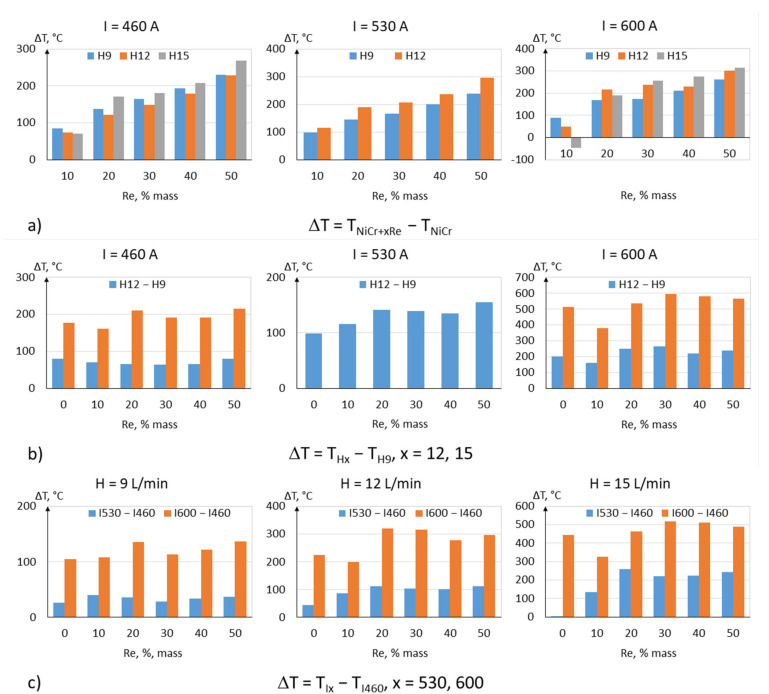
Changes in in-flight particle temperature ΔT: (**a**) in relation to rhenium content, (**b**) in relation to hydrogen flowrate, and (**c**) in relation to current intensity.

Based on the results discussed above, it can be concluded that modifying powders with rhenium allows for reaching in-flight temperatures of particles up to several hundred degrees Celsius higher, depending on the rhenium content and spraying parameters. The highest increase in temperature, about 300 °C, was achieved for powders with the highest rhenium content, as shown in Figure 14.

For a hydrogen flowrate of ν = 9, 12 L/min, none of the tested powders’ temperature reached the melting point of rhenium (3186 °C). However, in-flight particle temperature exceeded the Ni20Cr base powder melting point, which could promote the formation of Ni–Cr–Re alloys. For the highest hydrogen flowrate of ν = 15 L/min and a current intensity of I = 600 A, the in-flight temperature of particles exceeded rhenium’s melting point, which could also promote alloying between components, but also intensify evaporation effects.

It can be concluded from the obtained results that there are other factors that can greatly influence the in-flight temperatures of the particles, i.e., the process parameters (Figure 14b,c). At a constant current intensity and increasing hydrogen flowrate, the particle temperature can be increased by about 500 °C (Figure 14b). A similar effect can be achieved by increasing the torch current intensity (Figure 14c), in which case an increase in the in-flight temperature of around 500 °C was recorded.

**Figure 15:**
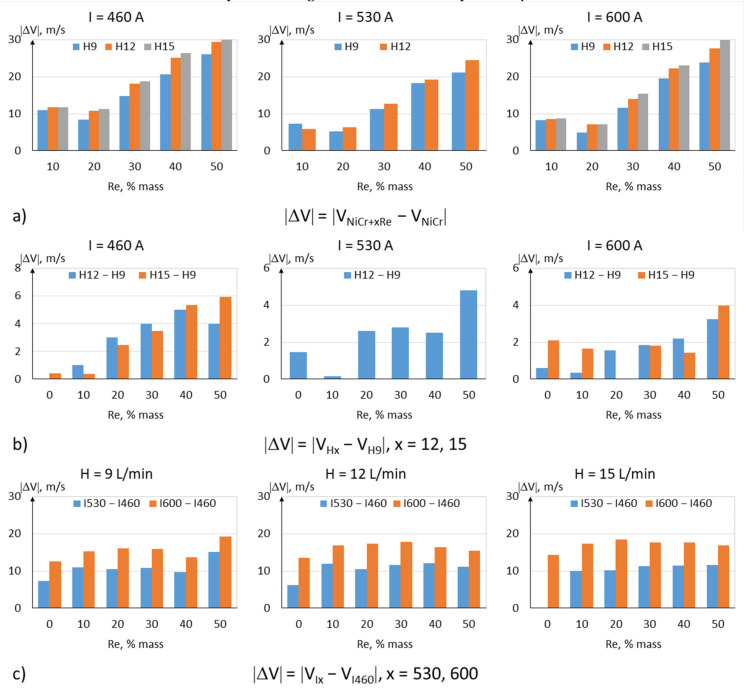
Changes in in-flight particle velocity |ΔV|: (**a**) in relation to rhenium content, (**b**) in relation to hydrogen flowrate, and (**c**) in relation to current intensity.

The Rhenium modification also influences the in-flight velocity of the particles—decreasing it. This is probably the result of the higher density of particles containing rhenium (Figure 13). The most significant decrease in velocity, on the order of 30 m/s, was recorded for powders with the highest rhenium content (Figure 15a). Taking into account a 2% measurement uncertainty, the plasma gas flowrate does not appear to influence the velocity of the particles. A slight increase in velocity, on the order of 20 m/s, can be achieved by increasing the current intensity of the plasma torch.

## 4. Discussion

Ni20Cr + xRe powders were successfully fabricated by the reduction of a mixture of Ni20Cr powder and ammonium perrhenate. However, chemical analysis found that there was some discrepancy between the targeted and actual content of rhenium in the powder. The reason could be the partial evaporation of rhenium oxides, which are highly volatile. Therefore, an appropriate overhead should be included when preparing such modified powders.

The XRD investigation of modified powders found some changes in cell parameters of the main phases. This may indicate some solid solution(s) formation in the material during the reduction process. Additionally, some minute contamination with mixed rhenium and nickel oxide, as well as its hydrate, might be present in the Ni20Cr + xRe powders.

SEM-EDS analysis indicates that modified powders consist of grains made of a Ni20Cr core that are covered in a rhenium shell. Supplementary EPMA investigations were made, and it was found, that rhenium diffuses into Ni20Cr alloy. Due to the low thickness of the rhenium shell, it was not possible to establish, if nickel or chromium diffused into rhenium. However, overall results seem to support the supposition that the solid solutions between the base alloy and the rhenium were made during the reduction. This supposition is also supported by the density of the modified powders, the values of which are closer to the theoretical density of the alloy of the used constituents than to their mixture.

When it comes to physical properties, rhenium modification increases the specific surface area, decreases the flowability, and results in lower d_10_, d_50_, and d_90_ values for particle size distribution. This can be the result of several factors—for example, comminution of the powder during co-milling base Ni20Cr and APR or the formation of fine rhenium particles that did not take part in creating shells on the base powder. While those changes are detrimental in general, obtained modified powders were of sufficient quality for use in plasma spraying technology.

The modification of Ni20Cr powder with rhenium strongly influences in-flight particle parameters. Generally, modified powders achieve a higher in-flight temperature and a lower in-flight velocity. Whether the increase in the in-flight temperature is only the direct result of a slightly longer residence time of the powder in the plasma plume due to its lower in-flight velocity is not clear. Some additional effects, such as the penetration of the powder to higher-temperature regions of the plasma plume due to the increase in density or different interactions of the modified powder with plasma (e.g., the lower evaporation of powder material) might play a role in in-flight temperature increase.

Regardless of the underlying mechanisms, the modification of the base powder with a refractory metal shell can lead to an increase in in-flight particle temperature of the powder, as predicted and calculated by researchers in Ref. [23]. The magnitude of the effect depends on the plasma spraying parameters—the torch current and the hydrogen flowrate.

## 5. Conclusions

Ni20Cr alloy powders were surface-modified with rhenium by the use of the thermoreduction method. Structural investigations revealed that interdiffusion between modified powder components led to the formation of an intermediary solid solution(s). The modification with rhenium affects the morphology of the base powder, increasing their specific surface area and decreasing the flowability. These changes do not prevent them from being used as feedstock material for the atmospheric plasma spraying process.

In-flight measurements validated the concept of coating nickel-based powders with a refractory metal to increase in-flight particle temperatures. The effect of lowering the particle speed of modified powders was also observed.

## Figures and Tables

**Table 1:** Base material properties—commercially available AMPERIT 250 (Ni20Cr) powder.

Name	Density, g/cm^3^	Flowability, s	Particle Size
AMPERIT 250	8.2973	27.6	d_10_ = 31.55
			d_50_ = 73.79
			d_90_ = 143.176

**Table 2:** The APS process parameters combinations.

Process Number	Current, A	Hydrogen Flowrate L/min	Argon Flowrate, L/min
1	460	9	54
2		12	
3		15	
4	530	9	
5		12	
6		15	
7	600	9	
8		12	
9		15	

**Table 3:** Rhenium content in Ni20Cr + xRe powders.

No.	Powder	Re Content, wt.%
1.	Ni20Cr + 10Re	9.4
2.	Ni20Cr + 20Re	18.6
3.	Ni20Cr + 30Re	28.0
4.	Ni20Cr + 40Re	38.0
5.	Ni20Cr + 50Re	48.0

**Table 4:** Results of EDS analysis of NiCr + 30Re powder surface. Point numbers correspond to points chosen for analysis, shown in Figure 5.

Point	Elemental Content, wt.%			
	O	Cr	Ni	Re
1	0.2	1.8	9.0	89.0
2	0.3	14.3	55.1	30.4

**Table 5:** Elemental composition of NiCr + 30Re powders investigated by the WDS method in areas shown in Figure 6.

Area No.	Area 1 (Figure 6b), wt.%			Area 2 (Figure 6c), wt.%		
	Ni	Cr	Re	Ni	Cr	Re
1	78.0	18.4	3.7	79.6	19.5	1.0
2	74.9	18.2	6.8	79.7	19.4	0.9
3	80.1	19.1	0.7	79.9	20.1	0.0
4	80.3	19.6	0.0	80.0	19.5	0.5
5	80.3	19.1	0.6	80.0	19.7	0.3
6	80.8	19.3	0.0	79.6	20.0	0.4
7	79.2	20.8	0.0	80.9	19.5	0.2
8	79.9	20.1	0.0	N/A	N/A	N/A

## Data Availability

Not applicable.
